# Psychological Factors as Determinants of Chronic Conditions: Clinical and Psychodynamic Advances

**DOI:** 10.3389/fpsyg.2021.635708

**Published:** 2021-01-28

**Authors:** Ciro Conversano, Mariagrazia Di Giuseppe

**Affiliations:** Department of Surgical, Medical and Molecular Pathology, Critical and Care Medicine, University of Pisa, Pisa, Italy

**Keywords:** chronic disease, personality, defense mechanisms, depression, alexitimia, psychosomatic, post-traumatic stress disorder

The involvement of psychological factors in the etiology of chronic diseases is rousing the interest of the scientific community (Conversano, 2019; Martino et al., 2019b; Merlo, 2019; Lenzo et al., 2020; Vicario et al., 2020), leading to an increase in research on neuropsychological correlates of a number of chronic illnesses including cardiovascular disease (Eikeseth et al., 2020), diabetes mellitus (Martino et al., 2019a), bone health (Catalano et al., 2018; Fiegl et al., 2019; Kelly et al., 2019, 2020; Lauriola et al., 2019; Williams et al., 2020), fibromyalgia (Veltri et al., 2012; Palagini et al., 2016; Conversano et al., 2019; Marchi et al., 2019), as well as neuropsychological problems such as attention and hyperactivity deficit (Fabio et al., 2018; Di Giuseppe et al., 2020c) and post-traumatic stress disorder (Carmassi et al., 2014, 2018; Settineri et al., 2018; Conversano et al., 2020a; Merlo et al., 2020; Orrù et al., 2020). A number of recent studies have demonstrated that personality traits and implicit emotion regulation are associated with development, progression, recurrence, and severity of chronic illness (Koole and Rothermund, 2011; Ciuluvica et al., 2019; Settineri et al., 2019; Rymarczyk et al., 2020). For instance, the adaptiveness of defense mechanisms determines greater quality of life, adherence to treatment, and improved survival rates in cancer patients (Porcerelli et al., 2017; Zimmerman et al., 2019), which suggests the need for the systematic assessment of defensive functioning in chronic diseases (Di Giuseppe et al., 2020b). Adverse childhood experiences are also risk factors for metabolic alterations and obesity (Pervanidou and Chrousos, 2012; Davis et al., 2014). In particular, research in clinical psychology demonstrates high comorbidity between cardio-metabolic diseases and major depressive disorder (MDD). The occurrence of stressful life events (SLEs) appears to be related to cardio-metabolic complications and comorbidities, as they directly affect life stress and compensatory behaviors (Rich-Edwards et al., 2012; Kessler and Bromet, 2013; Kesebir, 2014; Rock et al., 2014). Furthermore, the impact of traumatic events on well-being is associated with the nature, timing, duration, and course of the SLE (Phifer and Norris, 1989; Fisher et al., 2010). Adults who experienced their most distressing trauma in childhood exhibited more severe symptoms of PTSD and lower subjective happiness as compared to adults who experienced it in a later stage of development (Ogle et al., 2013). Research has demonstrated that higher serum triglyceride and lower HDL-cholesterol concentrations can be observed in depressed patients with SLEs as compared to depressed patients without SLEs (Péterfalvi et al., 2019). Moreover, high LDLC and low serum levels of HDL-C were found to be associated with physical and sexual abuse, whereas raised TG and lower HDL-C were found to be associated with childhood neglect and emotional abuse (Li et al., 2019). Cardio-metabolic diseases are also associated with poor performance and cognitive dysfunction in memory, attention, visuo-spatial abilities, and executive functions (Yaffe et al., 2009; Yates et al., 2012; Olson et al., 2017; Guicciardi et al., 2019; Wooten et al., 2019). Such a significant corpus of research has inspired reflection on how personality traits, defined as individual differences in characteristic patterns of thinking, feeling, and behaving (American Psychiatric Association, 2013), may affect the physical and psychological conditions of chronic patients.

Psychodynamic research has highlighted the role of personality in the development and progression of psychopathological and organic diseases (Price et al., 2001; Coughlin, 2011; Dell'Osso et al., 2012; Radziej et al., 2015; Boldrini et al., 2019; Catalano et al., 2019; Martino et al., 2020d). A number of studies have analyzed how personality characteristics may increase the risk of specific somatic diseases or the individual's general susceptibility to diseases (Friedman and Rosenman, 1959; Greer and Morris, 1975; Denollet et al., 1995; Horwood et al., 2015). Scholars have in recent years hypothesized that the occurrence of cancer is more frequent in individuals with cancer-prone personalities, also known as a Type C Personality (Eysenck, 1994; Watson et al., 1999; Lemogne et al., 2013). This hypothesis has been confirmed by research on defense mechanisms which has demonstrated that individuals who use mature defensive functioning, defined as the use of high-adaptive defensive strategies that lead the subject to the best adjustment and possible resolution of internal and external stressors, report higher physical and psychological functioning (Garssen, 2004; Paika et al., 2010; Petric et al., 2011; Perry et al., 2015; Di Giuseppe et al., 2019). Conversely, maladaptive defense style, defined as a combination of immature defensive strategies activated to keep the individual unaware of experiencing unmanageable feelings, desires, and thoughts, was shown to predict sleep disturbance, worse clinical conditions, and lower survival rates in cancer patients (Beresford et al., 2006; Hyphantis et al., 2011, 2013a,b; Hyphantis et al., 2016; Conversano et al., 2020c). In particular, the high use of repression leads to impairment of endocrine and immune functions and is common in patients with shorter disease-free intervals, shorter survival, and a more unfavorable cancer staging at endpoint (Bahnson and Bahnson, 1966; Kreitler et al., 1993; Weihs et al., 2000; Giese-Davis, 2008; Boscarino and Figley, 2009).

One aspect of personality commonly studied in patients with chronic diseases is alexithymia, which is defined as the inability to distinguish between emotions, thoughts, and physiological responses to stimuli. Alexithymia has been found to be associated with several medical conditions (Lumley et al., 2005; Willemsen et al., 2008; Honkalampi et al., 2010; Pouwer et al., 2010; Tolmunen et al., 2011; Mazaheri et al., 2012; Sapozhnikova et al., 2012; Shinkov et al., 2018). Alexithymia is associated with hyperarousal, physical symptoms, and unhealthy compulsive behaviors. Moreover, psychological treatments have poor outcomes in alexithymic patients, posing the question as to whether alexithymia can be improved through treatment (Lumley et al., 2007). Recent studies have found that alexithymic patients ranged from 25 to 50% among patients with Type 2 Diabetes Mellitus (Martino et al., 2020c) whereas this was not observed in patients with Inflammatory Bowel Disease (Martino et al., 2020b). This association between alexithymia and metabolic control was suggested by the negative correlation with HbA1c values. Since HbA1c reflects the mean serum glucose levels over time, it may be speculated that alexithymia may more probably be identified in patients with uncontrolled diabetes. Conversely, the attempt to restore euglycemia, in particular in subjects with high HbA1c and high serum glucose levels, may expose patients to hypoglycemic risk. Thus, the contribution of hypoglycemia, usually a manifestation of inadequately controlled diabetes, may not be ruled out. However, the study by Martino et al. was focused on a homogeneous T2DM population taking metformin and at relatively low hypoglycemic risk. Furthermore, alexithymia was found to be associated with anxiety and depression, especially in patients with poor compliance and adherence, concurring in a worse clinical picture and course of chronic diseases (Leweke et al., 2012; Hintistan et al., 2013; Mnif et al., 2014; Stanton and Hoyt, 2017; Rosa et al., 2019; Martino et al., 2020a).

Among other factors which contribute both to the onset and to the course of chronic illness, stressful life events are involved in the pathogenesis of both psychological and organic diseases (McFarlane, 2010; Afari et al., 2014; Marazziti et al., 2015). In addition, suffering from a chronic medical condition is a stressful factor *per se* and its influence on individual psychological well-being has been widely documented (Alonzo, 2000; Chaturvedi et al., 2017). Research has found that depression and psychosocial stressors promote inflammation and oxidative/nitrosative stress, decreased immunosurveillance and dysfunctional activation of the autonomic nervous system and of the hypothalamic-pituitaryadrenal axis (Piccinni et al., 2012; Bartolato et al., 2017). Accordingly, recent studies of the general population have demonstrated clinical levels of psychological distress, post-traumatic symptoms and somatization in response to the stressful condition of quarantining (Di Giuseppe et al., 2020d; Prout et al., 2020), confirming the effect of stress on physical and psychological well-being. Moreover, sociodemographic characteristics further contribute to increase hyperarousal and distress, with young people and women showing higher a prevalence of anxiety, depression and post-traumatic stress symptoms (Brooks et al., 2020; Conversano et al., 2020b) as well as a higher risk of developing chronic diseases (Abad-Díez et al., 2014; Holzer et al., 2017; Di Giuseppe et al., 2020a).

Taking these findings together, we assume that psychological and organic issues are intercorrelated and a comprehensive understanding of chronic medical conditions should consider all aspects of the illness (Yoo and Ryff, 2019). Effective therapy should be tailored to the needs of the patient, as suggested by personalized medicine. This approach promotes earlier diagnoses, risk assessment, and optimal treatments in order to ensure better patient care and lower costs (Vogenberg et al., 2010; Zilcha-Mano, 2020). In this perspective, psychotherapeutic interventions should be considered as an essential part of the treatment, since they are effective in reducing symptoms of psychological distress that, in turn, may affect disease progression and mortality (Lingiardi et al., 2010; Barrera and Spiegel, 2014; Salvatore et al., 2015; Gelo and Salvatore, 2016; Tanzilli et al., 2018; Perry et al., 2020; Yonatan-Leus et al., 2020). As suggested by Fonagy, we should use “*the opportunities provided by bioscience and computational psychiatry to creatively explore and assess the value of protocol-directed combinations of specific treatment components to address the key problems of individual patients*” (Fonagy, 2015).

## Author Contributions

CC and MD contributed in equal part to the development, conceptualization, literature review, and writing of the manuscript. All authors contributed to the article and approved the submitted version.

## Conflict of Interest

The authors declare that the research was conducted in the absence of any commercial or financial relationships that could be construed as a potential conflict of interest.
